# Reliable determination of pulse-shape instability in trains of ultrashort laser pulses using frequency-resolved optical gating

**DOI:** 10.1038/s41598-022-25193-3

**Published:** 2022-12-05

**Authors:** Rana Jafari, Soroush D. Khosravi, Rick Trebino

**Affiliations:** 1 School of Physics, Georgia Institute of Technology, 837 State Street NW, Atlanta, GA 30332 USA; 2 Mathematics & Physics Department, Queens University of Charlotte, 1900 Selwyn Ave, Charlotte, NC 28274 USA

**Keywords:** Optical physics, Optical techniques

## Abstract

We describe a reliable approach for determining the presence of pulse-shape instability in a train of ultrashort laser pulses. While frequency-resolved optical gating (FROG) has been shown to successfully perform this task by displaying a discrepancy between the measured and retrieved traces for unstable trains, it fails if its pulse-retrieval algorithm stagnates because algorithm stagnation and pulse-shape instability can be indistinguishable. So, a *non-stagnating* algorithm—even in the presence of instability—is required. The recently introduced Retrieved-Amplitude N-grid Algorithmic (RANA) approach has achieved extremely reliable (100%) pulse-retrieval in FROG for trains of *stable* pulse shapes, even in the presence of noise, and so is a promising candidate for an algorithm that can *definitively* distinguish stable and unstable pulse-shape trains. But it has not yet been considered for trains of pulses with pulse-shape instability. So, here, we investigate its performance for unstable trains of pulses with random pulse shapes. We consider trains of complex pulses measured by second-harmonic-generation FROG using the RANA approach and compare its performance to the well-known generalized-projections (GP) algorithm without the RANA enhancements. We show that the standard GP algorithm frequently fails to converge for such unstable pulse trains, yielding highly variable trace discrepancies. As a result, it is an unreliable indicator of instability. Using the RANA approach, on the other hand, we find zero stagnations, even for highly unstable pulse trains, and we conclude that FROG, coupled with the RANA approach, provides a highly reliable indicator of pulse-shape instability. It also provides a typical pulse length, spectral width, and time-bandwidth product, even in cases of instability.

## Introduction

Ultrashort laser pulses have a wide range of important applications across many fields. But their use in almost every application requires a stable pulse train, indeed, not merely in energy per pulse, but also in shape, that is, in intensity and phase evolution during the pulse. One such example is high-harmonic generation (HHG), which is the basis of many groundbreaking applications, like attosecond tabletop light sources^1^, ultra-high speed (opto) electronics^2^, and attosecond spectroscopy, especially time-resolved soft X-ray and EUV spectroscopies^3–5^ which require extreme stability of all of the above-mentioned pulse characteristics. In addition, ultrafast spectroscopic techniques^6–13^ have made the study of fundamental properties of matter, such as the electronic structure, bound and free electron dynamics, quantum coherence, transient photo-induced phenomena, and quantum spin possible, and they too require stable pulse trains in order to avoid inducing varying medium excitations on each pulse. The presence of shot-to-shot pulse-shape instability can be especially problematic when a result depends nonlinearly on the excitation pulses or when small temporal variations in the signal pulse are under investigation (for example, vibrational coherences). Pump lasers used to generate ultrashort pulses at uncommon wavelengths, such as optical parametric oscillators and amplifiers, must be very stable because these latter sources’ output pulses depend nonlinearly and hence sensitively on the input light pulse properties. Also, pump-laser stability is a critical factor for achieving stable supercontinuum in optical fibers, particularly hollow core fibers^14,15^. Ultrashort laser pulses are now also widely used in medical^16–20^ and industrial applications^21^ for surgical, imaging, and micro-fabrication purposes. Some examples are two photon scanning microscopy, ophthalmology, ultrafast optical coherence tomography (OCT), orthopedics, neuroscience, microfluidic devices manufacturing, and heart surgery. In all such applications and many more, instability is highly undesirable.

In general, pulse-shape instability is never desirable.

Unfortunately, ultrashort-laser pulses often suffer from this problem, caused by ambient and intrinsic factors such as temperature fluctuations, air turbulence, misalignment, unstable light-generation or amplification process, and partial mode locking, to name only a few of the many such causes. Instability is more likely to be present in laser systems with lower repetition rates and shorter pulses. And it is especially likely in novel laser systems that push the envelope of ultrashort-pulse technology, e.g., few-cycle-pulse or exotic-wavelength lasers.

The issue of pulse-to-pulse intensity-and-phase shape variations has plagued ultrashort-pulse lasers and their measurement from the beginning^22,23^. When confronted with a train of unstable pulse-intensity shapes, intensity autocorrelation yields a broad background under a narrow spike whose width corresponds to the coherent temporal component of the randomly varying pulses in the train. This spike, which is shorter than the average pulse and much shorter in the presence of pulse-shape instability, has come to be called the *coherence spike* or *coherent artifact.* Unfortunately, it provides a confusing indicator of instability when the instability is large, when the autocorrelation background is easily overlooked, and also when it is small, when the coherence spike blends into the pulse autocorrelation and hence yields an innocent-looking autocorrelation trace and an erroneously short pulse length.

Over time, the instability-detection problem has actually gotten worse, rather than better. It is now possible to measure the complete intensity and phase vs. time for an arbitrary pulse in a *stable* pulse train^24^, but many such techniques have yet to be examined for this issue, and there remains no “pulse-shape-stability measurement device” that can independently establish pulse-shape stability or instability. So, the task of determining the presence, magnitude, and type of such fluctuations necessarily falls to the pulse-measurement technique. And unfortunately, insufficient effort has been expended to solve this problem. Fortunately, recently^25–29^, the effect of pulse-shape instability in some pulse-intensity-and-phase-measurement techniques has been studied, and, unfortunately, some popular (interferometric) techniques were found to measure essentially *only* the coherence spike in the presence of pulse-shape instability. So, determining the stability of a pulse train remains an ongoing problem.

While single-shot measurement could potentially solve this problem, it is not usually possible to do this, so we must consider multi-shot measurements averaged over many, possibly quite different pulse shapes. For any technique, when instability is present, the measured trace is the sum of many different traces corresponding to the many different traces of the many different pulses. As a result, a measured trace of such a pulse train no longer corresponds to any single pulse. Consequently, discrepancies between the measured and retrieved frequency-resolved-optical-gating (FROG) traces, for example, are a good indicator of instability, especially in view of the fact that the FROG trace is an *N* × *N* array, used to measure only 2* N* parameters of the pulse, yielding significant overdetermination of the pulse. Specifically, a FROG trace with a coherence spike does not correspond to a pulse in FROG. For such traces, the FROG pulse-retrieval algorithm’s aim would be to find a pulse whose trace is closest to the *entire* measured trace, as such a result would, in some sense, represent the average features of the unstable train. In the above-mentioned studies of the effect of pulse-shape instability, it was established that FROG yields the approximately correct pulse length in essentially all cases when the algorithm converges to the trace closest to the measured trace^26^. However, the well-established simple generalized-projections FROG pulse-retrieval algorithm^30^, which is fairly reliable for stable pulse trains of even complex pulses, might not converge to the closest trace (that is, it may stagnate), yielding retrieved traces with higher discrepancies and retrieved fields that cannot be used to represent the train. This is because “convergence” to a trace that does not correspond to an actual pulse is considerably more difficult than convergence to a trace that does. Indeed, the term “convergence” is not yet a meaningful term in this case. We will remedy this problem later in this publication.

To reiterate, in the presence of instability, the FROG trace closest to the measured trace still necessarily shows disagreement with the measured trace, and it is this disagreement that is the indicator of instability. This disagreement, along with the retrieved field that represents the average features of the pulses in the train, such as the average rms time-bandwidth product (*TBP*_*rms*_), can be used to describe the pulses in the system. It should be noted that the recently introduced algorithms devised for separate measurement of average and coherent parts of unstable trains have their own drawbacks. For example, it was shown that, for the underlying algorithm for the pulse-retrieval step in the standard generalized projection (GP) algorithm, the same issue of stagnation may arise.^31^ Moreover, the additional assumption that the coherent component be a Fourier-transform-limited pulse does not allow the generalization of the algorithm, for example, to FROG traces of unstable double pulses or chirp instability.

The issue of algorithm stagnation is the key problem for the measurement of unstable pulse trains in any technique, including FROG. Stagnation and instability both yield discrepancies between the measured and retrieved traces and which one is the cause of a trace discrepancy could be difficult to identify. This could be especially problematic for moderately complex pulses when the standard GP algorithm is used, in view of its unreliability for such pulses. Indeed, for *stable* pulse trains, trace discrepancies due to stagnation could be confused for instability^32^. And when stagnation occurs for trains of *unstable* pulses, the extent of the instability would likely be misinterpreted as larger than is the case in reality. Consequently, the resulting retrieved pulse would be less accurate or even wrong.

To solve this problem, we have been developing a robust FROG pulse-retrieval algorithmic approach, the Retrieved-Amplitude N-grid Algorithmic (RANA) approach for common FROG variations^33–35^, and we have first tested it on stable pulse trains and have found it to be 100% reliable for stable complex pulse trains of pulses.

The RANA approach works in conjunction with any FROG algorithm. It first involves *directly* retrieving the spectrum from the FROG trace without additional measurements and then using it as the initial guess for the algorithm. Second, a set of a dozen or so initial guesses are generated, all with the directly retrieved spectrum, but with random noise for the spectral phase. Third, these initial guesses are first quickly run on smaller, coarser grids generated from the full trace^36,37^, removing the poorly performing pulses and keeping only the best-performing ones, which are then used for more computationally complex iterations involving the entire trace. When incorporating the well-known standard GP algorithm, RANA achieved convergence for 100% of tens of thousands of even extremely complex pulses with time-bandwidth products of up to 100 and even in the presence of significant noise in the measured trace.

However, RANA has not yet been tested for *unstable* pulse trains. As a result, in this work, we study the performance of the RANA approach for unstable pulse trains using SHG FROG. We show that RANA reliably converges to the field whose trace is closest to the measured trace—and the difference between the measured and retrieved trace corresponds entirely to pulse-shape instability and not algorithm stagnation. Therefore, it solves the problem of providing a pulse-train stability meter.

## What should we expect to measure?

In the presence of instability, no single correct spectrum exists. There will typically be a simple and smooth *average* spectrum, as would be measured, for example, by a spectrometer, which provides an erroneously short pulse and is *not* a desirable result. On the other hand, there will also be a potentially much more complex *typical* spectrum, which will usually be quite different. This is much more desirable.

A similar—but much more important—consideration is necessary for the spectral *phase*. A typical complex pulse’s spectral phase will, of course, be a complex curve. But, when the spectral phase is simply averaged over many such pulses, all with different random spectral phases, the result, like that for the average spectrum, will necessarily be much simpler. Indeed, it will usually be a simple *flat* spectral phase. And we should note that it is well-known that, for a given spectrum, the shortest pulse in time occurs for a flat (or linear) spectral phase^30,38^. Thus, the average spectral phase always yields a pulse shorter than—and potentially much shorter than—any of the actual pulses in the train. *In fact, a flat spectral phase is equivalent to the coherent artifact.* A flat spectral phase is simply the frequency-domain picture of the coherent artifact.

Thus, it is important that a pulse-measurement technique *not* yield the average spectral phase when instability is present. If it did, it would not be able to distinguish a stable train of short simple pulses (usually the best-case scenario) from an unstable train of long complex pulses (usually the worst-case scenario). This is an important and often misunderstood point, and several popular techniques in current use suffer from this problem^25–29^. So, it is important that the technique provide a spectral phase as close as possible to a *typical* spectral phase, which would then also yield the approximately correct average pulse length in time.

Having said this, we should also point out, however, that no SHG-based pulse-measurement technique can, to our knowledge, yield an accurate typical pulse in the presence of pulse-shape instability^25–29^. Only the third-order FROG techniques have been shown to be able to accomplish this, to our knowledge. Indeed, measurement devices, in general, yield an average measurement of the relevant quantities. And, in this work, as in previous studies of SHG FROG for measuring unstable pulse trains, we will find that we will need to settle for the typical pulse *length* and *TBP* when measuring unstable pulse trains. Nevertheless, we should mention that reliably determining the presence of instability and also the typical pulse length and TBP is still a nontrivial accomplishment for intensity-and-phase pulse-measurement devices, many of which generally yield only the average spectral phase (the coherent artifact) and hence a potentially much shorter pulse than is actually present on average. Worse, some also require an independently measured spectrum, obtained using a spectrometer, which, in addition, averages out any structure in the spectrum, contributing further to an anomalously short pulse.

## Simulations

**Figure 1:**
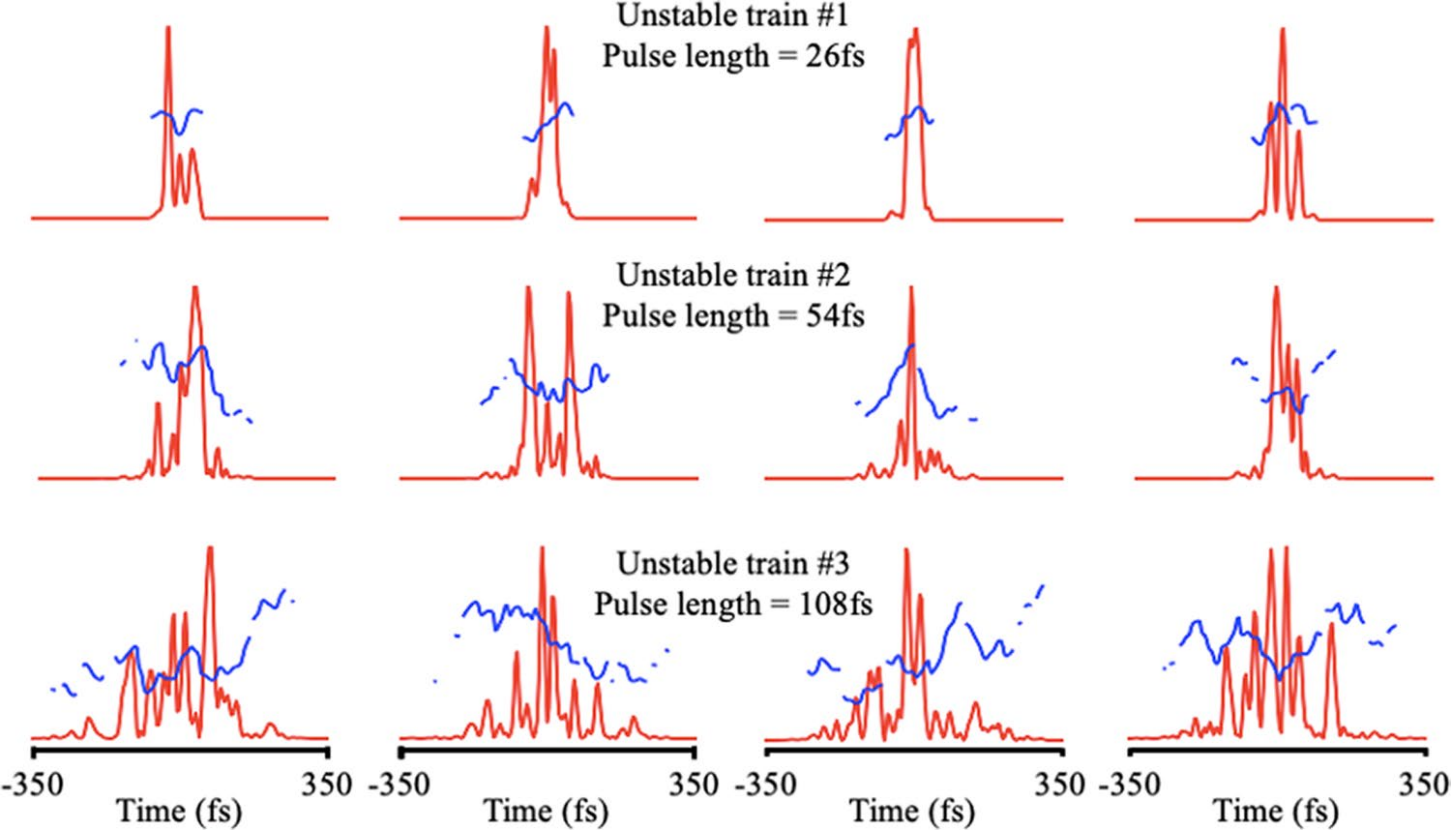
Samples of four pulses in the unstable trains with average FWHMs of 26, 54, and 108 fs, respectively (top to bottom). The temporal intensity and phase are shown by red and blue curves, respectively.

**Figure 2:**
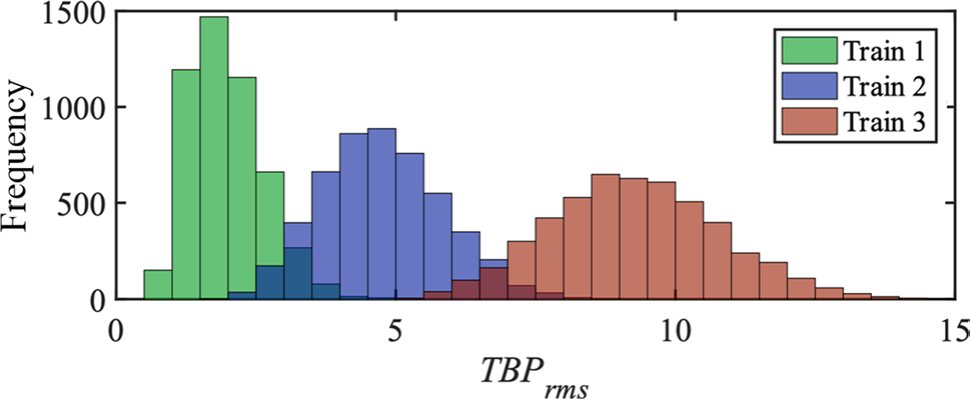
Distributions of the rms time-bandwidth product (*TBP*_*rms*_) of the 5000 pulses in the three simulated unstable trains: Train 1: *τ*_*FWHM*_ = 26 fs; train 2: *τ*_*FWHM*_ = 54 fs, and train 3: *τ*_*FWHM*_ = 108 fs.

We investigated the RANA approach’s performance on FROG traces corresponding to unstable pulse trains and compared the results with those obtained using the standard GP algorithm. For these simulations, we generated three pulse trains, each comprising 5000 different pulses. Each pulse consisted of a stable short pulse plus a longer unstable component using the method described in a previous publicaiton^26^. The pulse trains’ average properties were approximately those of the pulses in Ref.^25^, although, for this work, we also generated a more unstable train with an even longer average pulse length. The temporal full width at half-maximum (FWHM) of the nonrandom component was set to 12 fs. The random components had higher energies and were added to the stable train such that the average temporal FWHM of the pulses in the unstable trains became 26, 54, and 108 fs, respectively. Next, the energy of the pulses were adjusted to follow a normal distribution with a coefficient of variation (i.e., standard deviation over mean) of 10%. FROG traces were generated by averaging the individual traces of the pulses in a given train. Figure 1 depicts typical pulses in these trains, and Fig. 2 provides insight into the variations among the pulses for a given train by the *TBP*_*rms*_ distribution of the pulses in each train.

Due to the direction-of-time ambiguity of SHG traces, SHG FROG is, in some sense, the “weakest” version of FROG in terms of pulse-retrieval-algorithm performance^30^. But SHG FROG is the most popular FROG version due to its simple geometry and high sensitivity. For these two reasons, we only consider SHG FROG here and do not consider third-order versions of FROG; the latter methods are expected to exhibit better performance than SHG FROG and will be considered in a future publication.

We generated the relevant SHG FROG traces and retrieved pulses from each one of them for 100 different initial guesses using both the GP algorithm and the RANA approach (incorporating the GP algorithm). For traces corresponding to trains with average temporal FWHM of 26, 54, and 108 fs, traces with respective trace sizes of 128 × 128, 256 × 256, and 512 × 512 were used. The trace sizes were chosen so that the intensities at the perimeter of the trace drop to values smaller than 10^–4^ of the maximum intensity. Using both GP and RANA, we retrieved pulses from the three FROG traces generated for the three unstable trains and determined the *G*′ errors^39^ (the trace-intensity-area-normalized rms difference between the measured and retrieved traces), *TBP*_*rms*_, and the temporal FWHMs of retrieved fields. The maximum number of iterations for the standard GP algorithm and for the full trace step in the RANA approach was set to be 1500 and 375, respectively. These values are much higher than those we have used for stable pulse trains because, here, the traces no longer correspond to actual FROG traces, thus greatly complicating the multidimensional error surface and hence also the task of finding the best possible pulse. Since the algorithm fundamentally cannot reach extremely small *G-*error cutoff values used in typical theoretical studies, we terminated the iterations when the average of the absolute value of change between *G* error values (|*G*_*k*_ − *G*_*k*-1_|, where *k* is the iteration number) in the last ten iterations of the algorithm becomes less than 10^–7^. Note that the *G* error is the usual rms difference between the measured and retrieved traces, normalized by the number of pixels (it is irrelevant whether we used *G* or *G*′ for this condition).

In addition to retrievals from noise-free traces, we also considered traces with 3% additive and 5% multiplicative noise^40^. Noisy traces were pre-processed for both approaches in the usual manner, including background subtraction and Fourier filtering.

Because no single pulse corresponds to a FROG trace that has been distorted by pulse-train instability (this is good), no retrieved pulse can match the measured trace (also good). But this means that we must redefine what we mean by the term algorithm “convergence” in the presence of pulse-shape instability. Indeed, we must distinguish between measured-and-retrieved-trace discrepancies due to instability (good) and those of stagnation (bad). And even in the *absence* of instability, the GP algorithm can stagnate, and the final result could vary according to the initial guess. So, we define stagnation as occurring when either of the two common rms-difference measures of trace discrepancies, the *G* or *G*′ error values, are significantly higher than the lowest achievable *G* or *G*′ error. These latter numbers are obtained by running the relevant FROG algorithm(s) many times to find the smallest resulting value for *G* or *G*′. While this is cumbersome, this definition is only necessary for this study, and it is not practical or necessary or useful to use it in practice.

For this study, we compared the *G*′ errors as measure of the performance of both the GP and RANA approaches. In these simulations, the number of initial guesses and iterations on the smaller grids in the RANA approach were chosen based on the value of the *G*′ error (Table 1)^35^.

**Table 1:** Parameters used for trains with trace sizes 128 × 128, 256 × 256, and 512 × 512 (IG: initial guess).

*N*	# of IGs *N*/4 × *N*/4 array (RANA)	# of iterations *N*/4 × *N*/4 array (RANA)	# of IGs *N*/2 × *N*/2 array (RANA)	# of iterations *N*/2 × *N*/2 array (RANA)	# of IGs *N* × *N* array (RANA)	Minimum *G*′ error
128	32	45	20	40	4	~ 0.18
256	40	55	24	45	4	~ 0.22
512	56	55	32	45	4	~ 0.22

## Results

**Figure 3:**
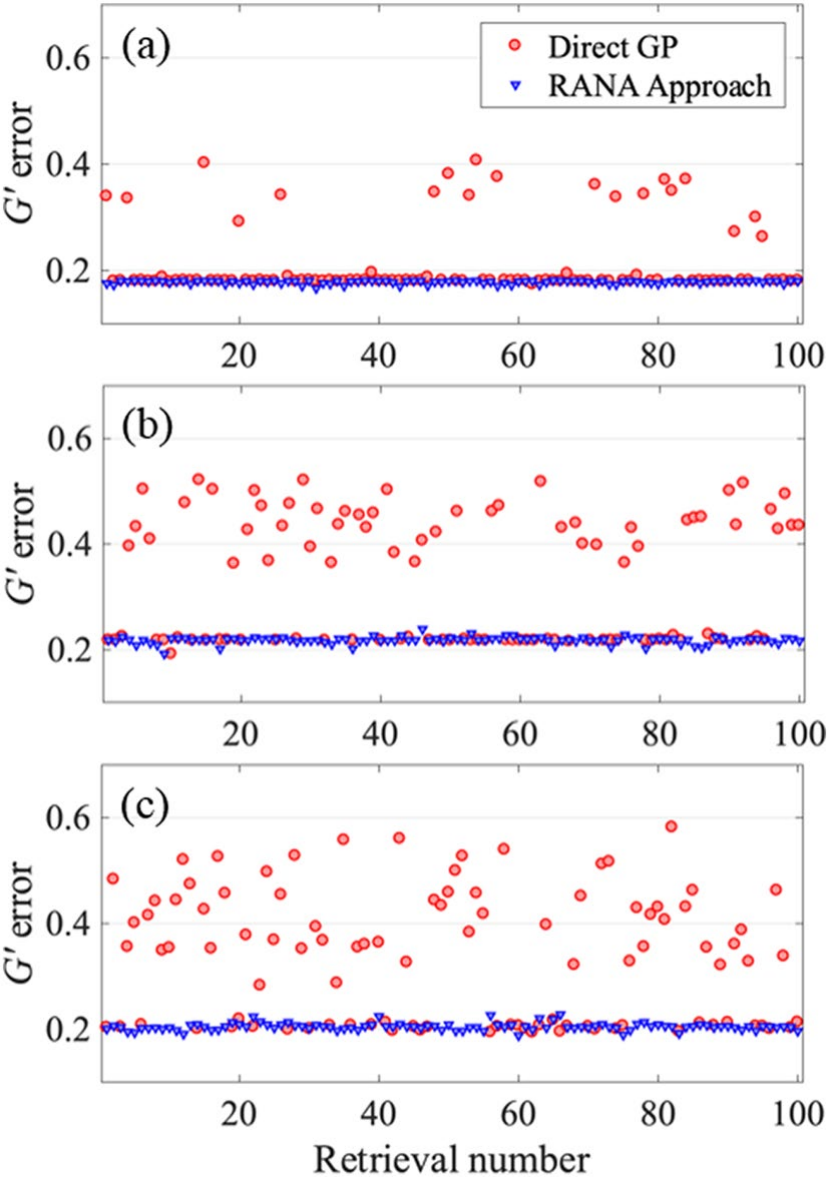
*G*′ errors for retrieval of three noise-free traces corresponding to three unstable trains with average temporal FWHM of 26 fs (**a**), 54 fs (**b**), and 108 fs (**c**). The *G*′ errors are obtained from the recovery of the pulse for 100 runs using the RANA approach and standard GP algorithm. Note that the RANA approach reliably yields the minimum *G*′ error, while the GP algorithm alone does not, and additional runs of the algorithm are required.

**Figure 4:**
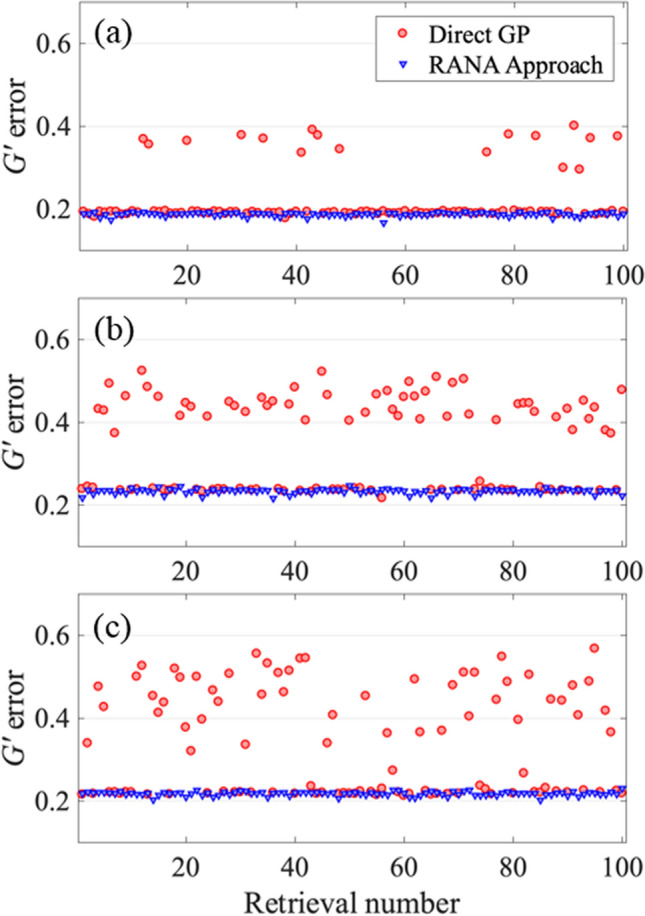
*G*′ errors for retrieval of three noisy traces contaminated with 3% additive and 5% multiplicative noise corresponding to three unstable trains with average temporal FWHM of 26 fs (**a**), 54 fs (**b**), and 108 fs (**c**). The *G*′ errors are obtained from recovery of the pulse for 100 runs using the RANA approach and standard GP algorithm from noise-reduced traces. Note that the RANA approach reliably yields the minimum *G*′ error, while the GP algorithm alone does not, and additional runs of the algorithm are required. Finally, note that the effects of trace noise are small compared to those of pulse-shape instability.

Figures 3 and 4 show the *G*′ errors for the standard GP algorithm and RANA approach for noise-free and noisy traces for 100 runs with different initial guesses. These results showed the RANA approach convergences to the field that yields a trace closest to the measured trace which corresponds to the field describing the average features of the train in all tries. On the other hand, the GP algorithm’s error varied significantly, and a few tries were generally needed to establish the minimum achievable *G*′ errors and choose the result with the lowest error.

For the train with average *τ*_*FWHM*_ = 26 fs, the GP algorithm converged to acceptable pulses with traces close to the measured trace for 81% (83%) of trials for noise-free (and noisy) traces, respectively. The performance of the standard GP algorithm decreased to 50% (47%) and 40% (48%) for the second and third trains with average *τ*_*FWHM*_ = 54 fs, and 108 fs, respectively. Note that the effects of trace noise were small compared to those of pulse-shape instability. In general, for a stable pulse train, the converged *G*′ error should equal the average noise in the trace measurement; converged *G*′ errors larger than the noise indicate pulse-train instability.

**Figure 5:**
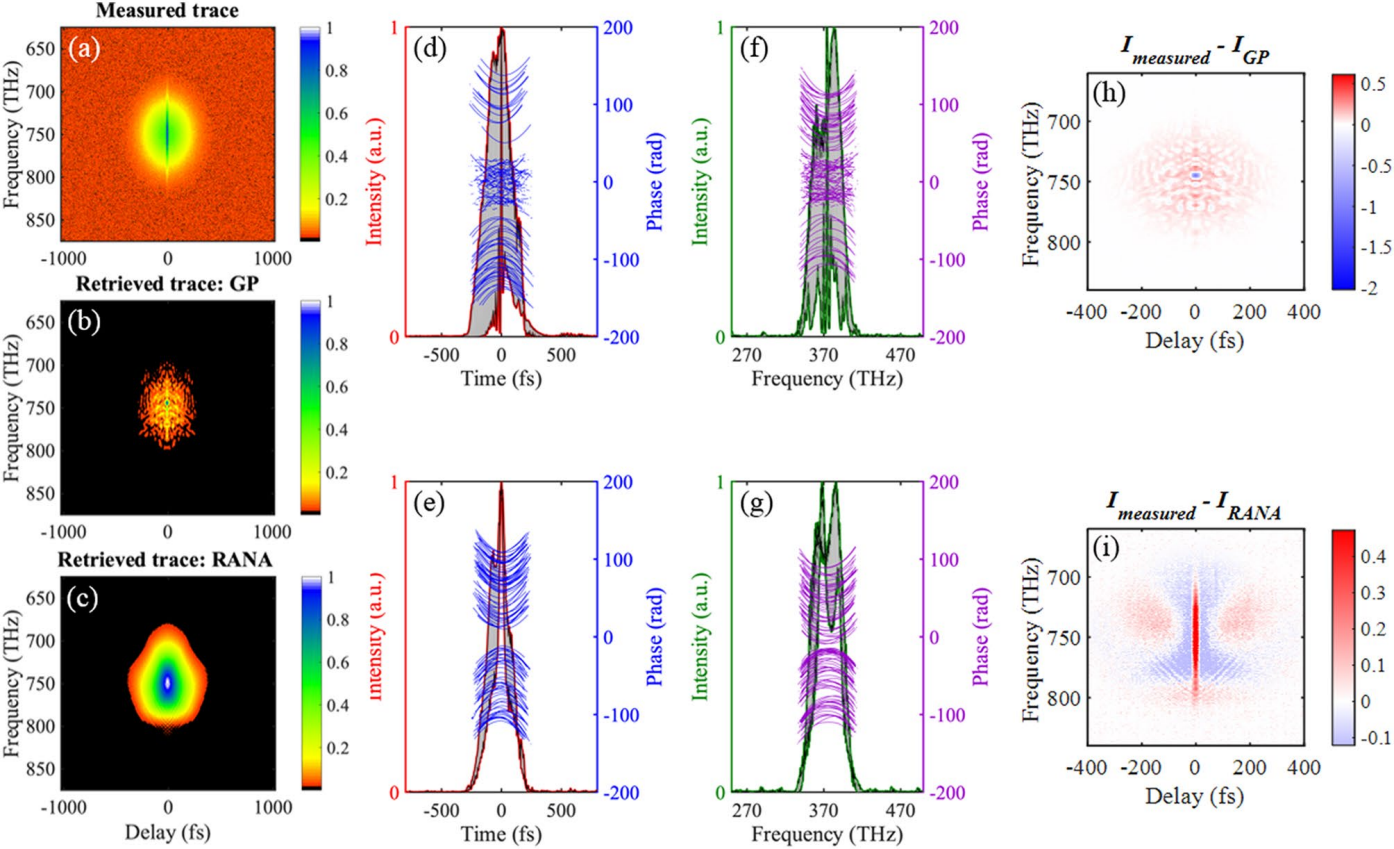
(**a**) The noisy trace corresponding to the train of pulses with an average temporal FWHM of 108 fs. Note the narrow coherent artifact for near-zero delays. (**b**) The reconstructed trace obtained using the GP algorithm for which the *G*′ error has the highest value (that is, the worst-case retrieval). (**c**) The analogous worst-case retrieved trace obtained using the RANA approach. The retrieved temporal and spectral intensities of the results corresponding to curves with maximum and minimum FWHMs and all the retrieved phases for GP (**d**,**f**) and the RANA approach (**e**,**g**) are shown. The phase curves grouped in the center of (**d**) and (**f**) correspond to the stagnated results, and the two other groups, with positive and negative chirps, are the retrieval results with the direction of time ambiguity inherent to SHG FROG, and correspond to results converged to the smallest achievable *G*′ errors. (**f**) The reconstructed trace obtained using the GP algorithm for which the *G*′ error has the highest value (that is, the worst-case retrieval). (**g**) The analogous worst-case retrieved trace obtained using the RANA approach. (**h**, and **i**) The discrepancy between the measured and retrieved traces for the RANA approach is entirely indicative of instability, whereas that for GP is partially due to stagnation.

Figure 5 shows all of the retrieved fields using the RANA approach and the GP algorithm from the noisy trace of train 3. It also shows the worst-case retrieved traces for both RANA and GP. Note that the standard GP algorithm retrieves a wide range of pulse shapes, depending on the initial guess, but some GP-retrieved pulses are not realistic and are the result of stagnation. Also, the worst-case (and many other unshown) GP-retrieved traces do not resemble the measured trace at all and so must be considered stagnations. The RANA approach-retrieved pulses, on the other hand, while simpler than the actual pulses, have pulse lengths close to those of the actual pulses.

The average of *G*′ errors and temporal and spectral widths of retrieved fields for the 100 retrievals from the three noise-free and noisy SHG traces are tabulated in Tables 2 and 3, respectively. The computed standard deviations of temporal and spectral FWHM show when convergence is not achieved, the retrieved fields yield features that vary significantly, and therefore, would not correspond to a meaningful and consistent result.

**Table 2:** The average *G*′ errors in 100 retrievals using the GP algorithm and RANA approach from noise-free SHG traces, and the temporal (*τ*_*FWHM*_) and spectral (*ω*_*FWHM*_) FWHM of all the retrieved fields.

	*G*′, GP	*G*′, RANA	*τ*_*FWHM*_ (fs), GP	*τ*_*FWHM*_ (fs), RANA	*ω*_*FWHM*_ (rad/fs), GP	*ω*_*FWHM*_ (rad/fs), RANA
Train #1	0.212 ± 0.066	0.178 ± 0.003	23.0 ± 4.7	24.4 ± 3.3	0.29 ± 0.09	0.34 ± 0.01
Train #2	0.331 ± 0.118	0.218 ± 0.006	43.0 ± 21.2	57.6 ± 8.5	0.20 ± 0.09	0.28 ± 0.01
Train #3	0.334 ± 0.120	0.205 ± 0.007	113.5 ± 60.0	157.7 ± 38.5	0.18 ± 0.07	0.25 ± 0.03

**Table 3:** The average *G*′ errors in 100 retrievals using the GP algorithm and RANA approach from noisy SHG traces, and the temporal (*τ*_*FWHM*_) and spectral (*ω*_*FWHM*_) FWHM of all the retrieved fields.

	*G*′, GP	*G*′, RANA	*τ*_*FWHM*_ (fs), GP	*τ*_*FWHM*_ (fs), RANA	*ω*_*FWHM*_ (rad/fs), GP	*ω*_*FWHM*_ (rad/fs), RANA
Train #1	0.220 ± 0.065	0.187 ± 0.004	22.9 ± 4.2	23.0 ± 2.7	0.28 ± 0.09	0.32 ± 0.01
Train #2	0.347 ± 0.107	0.233 ± 0.006	39.9 ± 23.6	56.8 ± 9.3	0.18 ± 0.09	0.27 ± 0.01
Train #3	0.339 ± 0.126	0.218 ± 0.005	109.5 ± 65.3	144.3 ± 36.0	0.18 ± 0.07	0.25 ± 0.02

For the more complex pulse trains, a coherent artifact can be clearly seen. This allows an alternative analysis^31^ that separates the trace into two components and solves for the coherent artifact separately. With this in mind, one could use the RANA approach to first confirm that a suspected spike is, in fact, a coherent artifact and then run the alternative analysis for more information about the coherent and incoherent components of the pulse.

**Figure 6:**
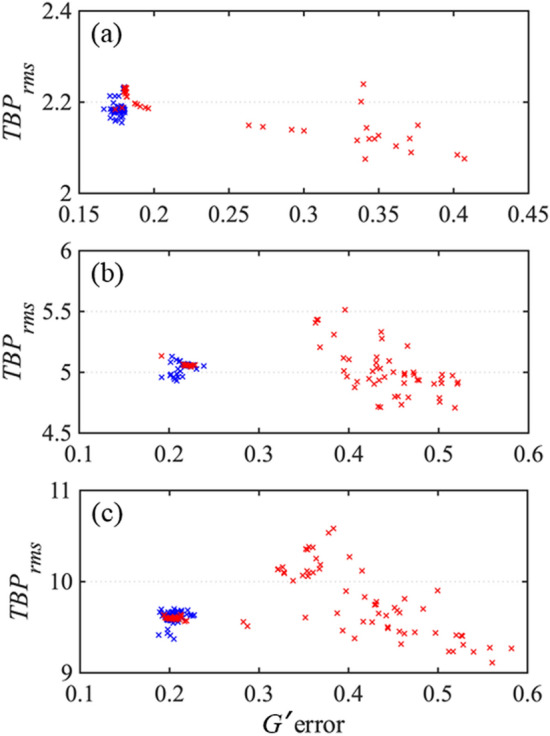
*TBP*_*rms*_ for the retrieved pulses using the RANA approach and the GP algorithm vs. *G*′ error are shown by blue and red crosses, respectively. The GP algorithm’s converged results have *TBP*_*rms*_ close to the average of the train, while the stagnated results have varying *TBP*_*rms*_, and show more variations as the pulse trains become more complex. The retrieved fields using the RANA approach are precise, consistent, and close to the average values for the pulses in the three trains with (**a**) *τ*_*FWHM*_ = 26 fs; *TBP*_*rms*_ = 1.97, (**b**) *τ*_FWHM_ = 54 fs; *TBP*_*rms*_ = 4.75, and (**c**) *τ*_FWHM_ = 108 fs; *TBP*_*rms*_ = 9.28. (In (**a**), an outlier for a retrieval result of the GP algorithm is not shown. The *TBP*_*rms*_ of the missing data is 4.6 with *G*′ error of 0.382).

Figure 6 depicts *TBP*_*rms*_ vs. *G*′ error for the 100 retrieved pulses using the GP and RANA approach from noise-free traces of three trains. The average values of *TBP*_*rms*_ for the fluctuating pulses of the trains with average *τ*_*FWHM*_ = 26, 54, and 108 fs were 1.97, 4.75, and 9.28, respectively. For the retrieval results from the RANA approach and the GP algorithm, where convergence is achieved, the *TBP*_*rms*_ are in good agreement with the actual values. From Fig. 6, it can be seen that if the stagnated results were used, the *TBP*_*rms*_ of the retrieved field would not necessarily correspond to the average value of the *TBP*_*rms*_ of the pulses in the train.

**Figure 7:**
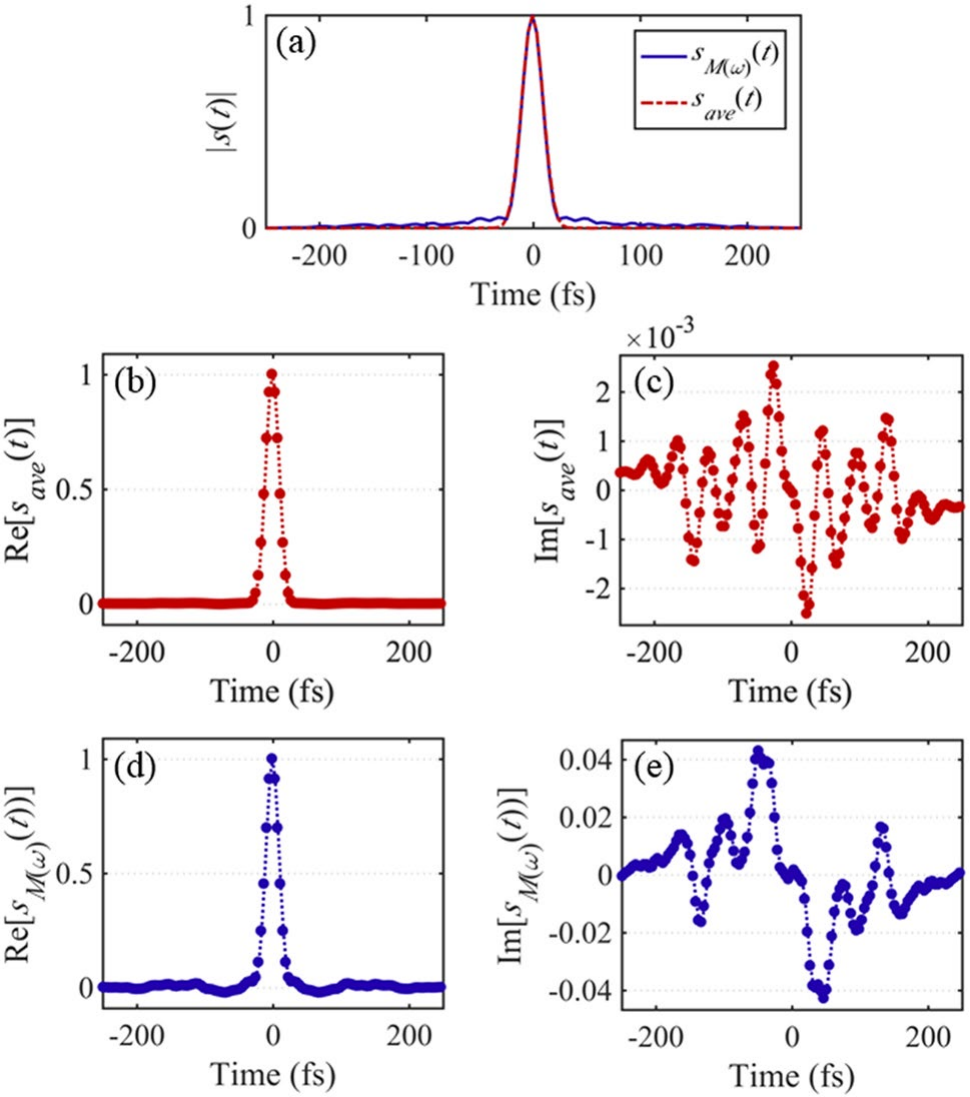
RANA approach spectral retrieval from the trace frequency marginal in the presence of instability. (**a**) The magnitudes of *s*_*ave*_(*t*) = F^−1^{*S*_*ave*_(*ω*)}, and *s*_*M*(_*ω*_)_(*t*) = √F^−1^{*M*^*SHG*^(*ω*)} corresponding to the train with *τ*_*FWHM*_ = 54 fs are plotted by the dashed red and the solid blue curves, respectively. (**b**, and **c**) Real and imaginary parts of *s*_*ave*_(*t*). (**d**, and **e**) Sign-ambiguity-removed curves of *s*_*M*(_*ω*_)_(*t*). As shown, there is an expected discrepancy between *s*_*ave*_(*t*) and *s*_*M*(_*ω*_)_(*t*). When instability is present, we do not expect to obtain the exact average spectrum from the frequency marginal. The above result is as good an estimate of an individual pulse spectrum as can be expected and turns out to be a good initial guess for the retrieval algorithm.

To examine the direct spectral retrieval step of the RANA approach for traces distorted by pulse-shape instability, we checked the difference between the inverse Fourier-transform of the average spectrum of the train, *s*_*ave*_(*t*) = F^−1^{*S*_*ave*_(*ω*)}^35^, as well as the square-root of the inverse Fourier-transform of the frequency marginal, *s*_*M*(_*ω*_)_(*t*) = √F^−1^{*M*^*SHG*^(*ω*)} as described in Ref.^33^. As an example, the magnitude of *s*_*ave*_(*t*), and *s*_*M*(_*ω*_)_(*t*) obtained from the second train are shown in Fig. 7. As is shown, these two curves do not match exactly, and there are discrepancies in both the real and imaginary parts of these two quantities due to the distortions in the trace induced by the instability.

**Figure 8:**
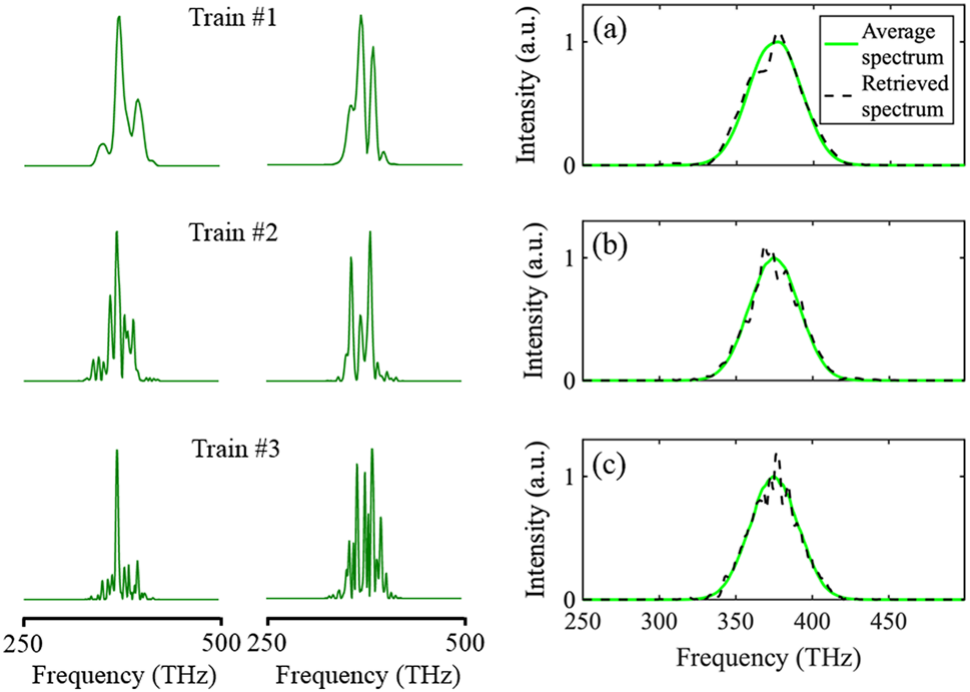
First and second columns: Typical spectra for trains 1 to 3 (top to bottom). Third column: The directly retrieved spectra from the marginal of the FROG trace, and the average spectra (as would be measured by a spectrometer averaging over all pulses in the train) for the three trains with average (**a**) *τ*_*FWHM*_ = 26 fs, (**b**) *τ*_*FWHM*_ = 54 fs, and (**c**) *τ*_*FWHM*_ = 108 fs. The discrepancy between these two spectra increases as the instability increases in the train, which is caused by the instability-induced deviation of the frequency-marginal curve from the autoconvolution of the average spectrum of the trains. Indeed, the spectral structure shown here is indicative of the structure actually present in a typical pulse in each train and so is a better estimate of the typical pulse spectrum than the average spectrum.

As discussed in Ref.^35^, four directly retrieved spectra are used as the spectra of initial guesses for the RANA approach in SHG FROG. Figure 8 shows the first choice among these four retrieved spectra from the marginal along with the average spectrum of the trace. It should be noted that the directly retrieved spectra are merely used as initial guesses for the algorithm, and the mismatch indicates the structure present in each pulse spectrum. It should also be noted that the average retrieval times for the traces containing instability are much higher than the corresponding times for stable trains. The reason for this is that the rate of convergence is much slower when the trace does not correspond to that of an actual pulse, and therefore more iterations are required for the algorithm to converge. Additionally, the RANA approach’s average retrieval time is smaller than the average time for the GP algorithm’s converging cases.

We also tested the RANA approach on a train of unstable double-pulses with fixed separation but a random relative phase between the double pulses. Both the GP and RANA approach converged to the low *G*′ error values for all 100 cases. For the case of unstable double pulsing with random relative phase and separation, both methods also reliably converged to the lower average *G*′ errors.

## Conclusion

In this work, we investigated the convergence behavior of the standard GP algorithm and the new RANA approach when provided with SHG FROG traces averaged over trains of pulses with unstable pulse shapes. We find that, when the usual GP algorithm is used for retrieval of pulses from FROG traces of such trains, the discrepancies between measured and retrieved traces are not always entirely due to the instability artifacts. Stagnation of the algorithm could also occur and contribute to the discrepancies, yielding additional undesired discrepancy between the measured and retrieved traces. This would complicate any attempt to identify the instability, and the retrieval could be performed a number of times, so that the result with the lowest *G* error could be used to represent the train. But even then, it would never be possible to know when to stop trying new initial guesses and to conclude that the discrepancy is due to instability and not algorithm stagnation. As a result, using this algorithm (or others that are not 100% reliable), it is not possible to definitively establish the presence of instability because the retrieved pulse can vary significantly according to the initial guess provided.

On the other hand, we find that the recently introduced RANA approach—the first 100% reliable pulse-retrieval algorithm for FROG for stable pulses—is also highly effective for determining the stability or instability of a pulse train. Our results indicate that, when a trace corresponding to a train of unstable pulse shapes, that is, with a coherent artifact, is measured, the RANA-retrieved trace consistently yields the least achievable discrepancy with respect to the measured trace (i.e., the lowest achievable value of *G*′ error or its close neighborhood at which point the algorithm is terminated). This consistent convergence establishes that, when the RANA approach is used for the retrieval of a pulse from such a trace, the final result corresponds to the best-retrieved typical field, and any disagreement between the measured and retrieved traces reliably implies pulse-shape fluctuations between pulses—and not algorithm stagnation. Also, the retrieved field has a pulse length and *TBP* close to the average value of the pulses in the train and is the best available representation of a typical pulse in the train, albeit with less structure than the typical pulse.

Thus, the RANA approach provides the desired reliability for, not only potentially complex pulses, but also for both stable and unstable pulse trains, and, as a result, can be used as a dependable indicator of the average pulse length, *TBP*, and pulse-train stability or instability.

## Data Availability

Data underlying the results presented in this paper are not publicly available at this time but may be obtained from the authors upon reasonable request.
